# Behavioral Effects of the Benzodiazepine-Positive Allosteric Modulator SH-053-2’F-S-CH_3_ in an Immune-Mediated Neurodevelopmental Disruption Model

**DOI:** 10.1093/ijnp/pyu055

**Published:** 2015-01-29

**Authors:** Juliet Richetto, Marie A. Labouesse, Michael M. Poe, James M. Cook, Anthony A. Grace, Marco A. Riva, Urs Meyer

**Affiliations:** Center of Neuropharmacology, Dipartimento di Scienze Farmacologiche e Biomolecolari, Università degli Studi di Milano, Milan, Italy (Drs Richetto and Riva); Physiology and Behavior Laboratory, ETH Zurich, Schorenstrasse 16, 8603 Schwerzenbach, Switzerland (Drs Labouesse and Meyer); Department of Chemistry and Biochemistry, University of Wisconsin–Milwaukee, Milwaukee, WI (Drs Poe and Cook); Departments of Neuroscience, Psychiatry and Psychology, University of Pittsburgh, Pittsburgh, PA (Dr Grace); Center of Excellence on Neurodegenerative Diseases, Università degli Studi di Milano, Milan, Italy (Dr Riva).

**Keywords:** autism, GABA, infection, poly(I:C), schizophrenia

## Abstract

**Background:**

Impaired γ-aminobutyric acid (GABA) signaling may contribute to the emergence of cognitive deficits and subcortical dopaminergic hyperactivity in patients with schizophrenia and related psychotic disorders. Against this background, it has been proposed that pharmacological interventions targeting GABAergic dysfunctions may prove useful in correcting such cognitive impairments and dopaminergic imbalances.

**Methods:**

Here, we explored possible beneficial effects of the benzodiazepine-positive allosteric modulator SH-053-2’F-S-CH_3_, with partial selectivity at the α2, α3, and α5 subunits of the GABA_A_ receptor in an immune-mediated neurodevelopmental disruption model. The model is based on prenatal administration of the viral mimetic polyriboinosinic-polyribocytidilic acid [poly(I:C)] in mice, which is known to capture various GABAergic, dopamine-related, and cognitive abnormalities implicated in schizophrenia and related disorders.

**Results:**

Real-time polymerase chain reaction analyses confirmed the expected alterations in GABA_A_ receptor α subunit gene expression in the medial prefrontal cortices and ventral hippocampi of adult poly(I:C) offspring relative to control offspring. Systemic administration of SH-053-2’F-S-CH_3_ failed to normalize the poly(I:C)-induced deficits in working memory and social interaction, but instead impaired performance in these cognitive and behavioral domains both in control and poly(I:C) offspring. In contrast, SH-053-2’F-S-CH_3_ was highly effective in mitigating the poly(I:C)-induced amphetamine hypersensitivity phenotype without causing side effects in control offspring.

**Conclusions:**

Our preclinical data suggest that benzodiazepine-like positive allosteric modulators with activity at the α2, α3, and α5 subunits of the GABA_A_ receptor may be particularly useful in correcting pathological overactivity of the dopaminergic system, but they may be ineffective in targeting multiple pathological domains that involve the co-existence of psychotic, social, and cognitive dysfunctions.

## Introduction

The central γ-aminobutyric acid (GABA) system is strongly implicated in cognitive processes. Accumulating evidence suggests that GABAergic interneurons critically regulate neuronal oscillatory activity (Gonzalez-Burgos and Lewis, 2012), which in turn is believed to serve various complex functions, including perception, cognition, and memory (Gonzalez-Burgos et al., 2011; Lewis et al., 2012). On these bases, various cognitive deficits found in psychiatric disorders with neurodevelopmental components may, at least in part, stem from a dysregulated inhibitory GABAergic interneuron network (Gonzalez-Burgos et al., 2011; Lewis et al., 2012; Volk and Lewis, 2013). Altered pre- and post-synaptic markers of cortical and hippocampal GABAergic neurotransmission are, in fact, among the most consistently observed abnormalities in developmental psychiatric disorders, most notably schizophrenia (Benes and Berretta, 2001; Volk and Lewis, 2013). Post-mortem studies conducted in schizophrenic patients report reduced expression levels of specific GABAergic interneuron markers, including parvalbumin and somatostatin (Hashimoto, Arion, et al., 2008; Morris et al., 2008; Fung et al., 2010; Konradi et al., 2011), along with deficient expression of various presynaptic regulators of GABA neurotransmission such as the 67 kDA isoform of the GABA synthesizing enzyme glutamic acid decarboxylase (GAD_67_) and the GABA transporter 1 (Hashimoto, Bazmi, et al., 2008). These changes are further accompanied by altered levels of GABA_A_ receptor subunits, including increased α2 subunits and decreased α1, α4, and α5 subunits in cortical layers of patients with schizophrenia (Hashimoto, Arion, et al., 2008; Hashimoto, Bazmi, et al., 2008; Duncan et al., 2010; Beneyto et al., 2011).

In addition to their involvement in cognitive processes, GABA-mediated inhibitory networks are also believed to critically regulate subcortical dopaminergic functions. According to a recent hypothesis (Lodge and Grace, 2011; Grace, 2012), disinhibition of the (ventral) hippocampus resulting from intrahippocampal impairments in GABAergic signaling could lead to a pathological hyperactivity of the (ventral) hippocampus and subsequent increase in mesoaccumbal dopamine system function. Such hippocampal abnormalities and states of subcortical hyperdopaminergia are prominent features of schizophrenia and related psychotic disorders (Zierhut et al., 2010; Lodge and Grace, 2011).

Against these backgrounds, it has been proposed that pharmacological interventions targeting GABA abnormalities may prove useful in correcting both cognitive impairments and dopaminergic dysfunctions present in patients with schizophrenia (Guidotti et al., 2005; Stan and Lewis, 2012). A first line of evidence supporting this hypothesis was derived from a small placebo-controlled clinical trial suggesting that treatment with a benzodiazepine-like agent with preferential activity at the α2/α3 subunit of GABA_A_ receptors can improve cognitive and electrophysiological measures of prefrontal functions in individuals with chronic schizophrenia (Lewis et al., 2008). Such pro-cognitive effects associated with positive allosteric modulation of the α2/α3 subunit, however, could not be replicated in a larger randomized clinical trial (Buchanan et al., 2011). In contrast, a novel α5 GABA_A_ receptor-positive allosteric modulator has been shown to reverse hyperactivation of the dopamine system in the methylazoxymethanol acetate (MAM)-based neurodevelopmental disruption model of schizophrenia (Gill et al., 2011), indicating that such GABAergic modulation may be useful in targeting positive symptoms of schizophrenic disease.

In the present study, we used an established immune-mediated neurodevelopmental disruption model to test the behavioral effects of SH-053-2’F-S-CH_3_, a relatively novel benzodiazepine positive allosteric modulator (PAM) with partial selectivity at the α2, α3, and α5 subunits (Fischer et al., 2010; Savić et al., 2010). The chosen model is based on prenatal administration of the viral mimetic polyriboinosinic-polyribocytidilic acid [poly(I:C)] in mice, which is known to capture a wide spectrum of behavioral and cognitive abnormalities relevant to neurodevelopmental psychiatric disorders (reviewed in Meyer and Feldon, 2010; Meyer, 2014). The prenatal poly(I:C) model has been established in relation to epidemiological studies showing increased risk of schizophrenia and related disorders following prenatal maternal exposure to infection or inflammation (Brown and Derkits, 2010). Importantly, prenatal poly(I:C) treatment in mice is capable of inducing a wide range of schizophrenia-relevant prefrontal and hippocampal GABAergic abnormalities in adult offspring, including reduced mRNA and/or protein expression of GAD_65_, GAD_67,_ and parvalbumin (Meyer et al., 2008; Piontkewitz et al., 2012; Richetto et al, 2013, 2014). Mice exposed prenatally to poly(I:C) also show diminished prefrontal expression of the α4 and α5 subunits of GABA_A_ receptors (Richetto et al., 2014) and increased α2-GABA_A_ receptor immunoreactivity at axon initial segments (Nyffeler et al., 2006; Meyer et al., 2008), akin to post-mortem findings in schizophrenia (Volk et al., 2002; Hashimoto, Arion, et al., 2008; Beneyto et al., 2011). These GABAergic changes are further paralleled by schizophrenia-relevant behavioral and cognitive dysfunctions, including impaired working memory and cognitive flexibility, reduced social approach behavior, and increased amphetamine (AMPH) sensitivity (Zuckerman et al., 2003; Meyer et al., 2005, 2008; Bitanihirwe, Peleg-Raibstein, et al., 2010; Bitanihirwe, Weber, et al. 2010; Connor et al., 2012; Richetto et al., 2013, 2014). Based on these findings, we tested whether positive allosteric modulation of the α2, α3, and α5 GABA_A_ receptor subunits by systemic SH-053-2’F-S-CH_3_ treatment may mitigate working memory deficiency, social interaction deficits, and AMPH hypersensitivity in adult offspring prenatally exposed to poly(I:C).

## Methods

### Animals

C57BL6/N mice were used throughout the study. This mouse strain was chosen based on our previous work investigating the long-term consequences of prenatal poly(I:C) treatment in mice (e.g., Giovanoli et al., 2014). Female and male breeders were obtained from our in-house, specific pathogen–free colony at the age of 12–14 weeks. Breeding began after 2 weeks of acclimatization to the new animal holding room, which was a temperature- and humidity-controlled (21±11°C and 55±5%, respectively) holding facility under a reversed 12h light and 12h dark cycle (lights off at 08:00h). All animals had *ad libitum* access to standard rodent chow (Kliba 3430, Provimi Kliba) and water throughout the study. All procedures described in the present study had been previously approved by the Cantonal Veterinarian’s Office of Zurich.

### Maternal Immune Activation During Pregnancy

Female mice were subjected to a timed mating procedure as described previously (Meyer et al., 2005). Pregnant dams on gestation day 17 (GD 17) received either a single injection of poly(I:C) (potassium salt; Sigma-Aldrich) at a dose of 5mg/kg or vehicle (VEH; sterile pyrogen-free 0.9% NaCl) according to protocols established before (Meyer et al., 2008; Bitanihirwe, Peleg-Raibstein, et al., 2010: Bitanihirwe, Weber, et al., 2010; Richetto et al., 2013, 2014; see also Supplementary Figure 1). The late gestational stage (i.e., GD 17) was selected because of our previous findings showing that GD17 poly(I:C) treatment is capable of inducing working memory deficiency and various pre- and postsynaptic GABAergic abnormalities in adulthood (Meyer et al., 2008; Richetto et al., 2013, 2014). Poly(I:C) was dissolved in sterile pyrogen-free 0.9% NaCl (VEH) solution to yield a final concentration of 1.0mg/ml and was administered via the intravenous (i.v) route at the tail vein under mild physical constraint as fully described elsewhere (Meyer et al., 2005). All solutions were freshly prepared on the day of administration and injected with a volume of 5ml/kg.

### Allocation and Testing of Offspring

All offspring were weaned and sexed at postnatal day 21 (Supplementary Figure 1). Littermates of the same sex were caged separately and maintained in groups of 2–4 animals per cage as described above. Only male mice were included in all experiments, to circumvent bias arising from sexual dimorphism. A first cohort of behaviorally naïve male animals comprising of 11 control (CON) and 9 poly(I:C) (POL) offspring was allocated to gene expression analyses of GABA_A_ receptor subunits in prefrontal and hippocampal tissues (see below). A second cohort of male animals comprising of 34 CON and 34 POL offspring was assigned to the behavioral and cognitive testing (Supplementary Figure 1), which included (1) spatial working memory in the Y-maze, (2) social approach and cognition, and (3) AMPH sensitivity tests (see below). To minimize the number of animals required to complete all tests of interest, mice were repeatedly tested, starting with the spatial working memory test and ending with the AMPH sensitivity test. A resting period of at least 1 week was inserted between each of these tests so as to allow sufficient drug wash-out from one test to another (Supplementary Figure 1). Previous drug histories (i.e., SH-053-2’F-S-CH_3_ or VEH treatment, see below) were counterbalanced across the prenatal treatment conditions from one test to another in order to further minimize possible confounds from drug carry-over effects. CON and POL offspring in both cohorts used in this study stemmed from multiple independent litters (19 CON litters and 18 POL litters) in order to avoid litter effects (Zorrilla 1997). Hence, 1–2 offspring per litter were used either for the gene expression analyses or the behavioral and cognitive testing.

The behavioral testing was always conducted during the animals’ active phase (i.e., during the dark phase of the reversed light-dark cycle between 09:00 and 18:00h). The time of daily testing was counterbalanced across the different experimental groups. To facilitate the visualization of spatial cues and to enable adequate video recordings, behavioral testing required that the animals were briefly exposed to a dimly-lit room. All experiments were conducted once the offspring reached the adult stage of development (i.e., from 14 weeks of age onwards).

### Gene Expression Analysis by Quantitative Real-Time PCR

Fresh brain tissue was collected and prepared for gene expression analysis by quantitative real-time polymerase chain reaction (PCR) as fully described in the Supplementary Materials. Following total RNA extraction, the samples were processed for real-time PCR to assess the five different α subunits (α1–α5) of GABA_A_ receptors using protocols established previously (Guidotti et al., 2012; Richetto et al., 2013, 2014; see also Supplementary Materials). Probe and primer sequences were purchased from Eurofins MWG-Operon and are summarized in Table 1. Relative target gene expression was calculated according to the 2(-Delta Delta C[T]) method (Livak and Schmittgen, 2001), and the data was expressed and analyzed as percentage of mRNA levels in control offspring. Gene expression was examined in the medial prefrontal cortex (mPFC; including the anterior cingulate, prelimbic, and infralimbic subregions; bregma: +2.3 to +1.3mm), striatum (Str; including dorsomedial and lateral caudate putamen; bregma +1.5 to +0.5mm), dorsal hippocampus (dHPC; bregma −1.5 to −2.5mm), and ventral hippocampus (vHPC; bregma −2.5 to −3.5mm).

**Table 1.** Primer Sequences Used in the Real-Time Polymerase Chain Reaction Analyses.

Gene	Forward Primer	Reverse Primer	Probe
**α1**	5’-TGAGAGCTGAATGCCCAATG-3’	5’-TCTGCTACAACCACTGAACG-3’	5’-CCTGCCCACTAAAATTCGGAAGCTATGC-3’
**α2**	5’-CCATGAGGCTTACAGTCCAAG-3’	5’-ACGGAGTCAGAAGCATTGTAAG-3’	5’-CGTAGCTTCCAAATTTCAGTGGGCA-3’
**α3**	5’-ACAATATGACCACACCCAACA-3’	5’-AGCTTCCAAACTTCAGTGGG-3’	5’-CAATACACGCTGAATGCCCCATGC-3’
**α4**	5’-GCCTGCCCTTTGAAATTTGG-3’	5’-GATACAGTCTGCCCAATGAGG-3’	5’-ATCTACACCTGGACCAAAGGCCC-3’
**α5**	5’-GGGAATGGACAATGGAATGC-3’	5’-TGTCATTGGTCTCGTCTTGTAC-3’	5’-CATTTGCGAAAAGCCAAAGTGACTGGA-3’
***36B4***	5’-AGATGCAGCAGATCCGCAT-3’	5’-GTTCTTGCCCATCAGCACC-3’	5’-CGCTCCGAGGGAAGGCCG-3’

### Working Memory in a Spatial Novelty Preference Y-Maze Paradigm

Working memory was tested using a spatial novelty preference task in the Y-maze as established before (Bitanihirwe, Weber, et al., 2010). The spatial novelty preference test in the Y-maze assesses spatial working memory and uses the natural tendency of rodents to explore novel over familiar spatial environments (Dellu et al., 1992). The apparatus and test protocol are fully described in the Supplementary Materials.

### Social Interaction Test

The test of social interaction was performed in a modified Y-maze as fully described in the Supplementary Materials. The test consisted of two phases, namely the dummy phase and the novelty phase, which measured social approach behavior and social recognition, respectively. During the first phase (dummy phase), the test mouse could freely explore an unfamiliar C57BL6/N mouse (live mouse) and an inanimate object (dummy mouse). The percent of time spent with the live mouse was calculated by the formula (time spent with the live mouse/(time spent with the live mouse + time spent with the dummy object)) × 100 and was used to assess relative exploration time between a congenic mouse and an inanimate dummy object. During the second phase (novelty phase), the test mouse could freely explore another unfamiliar C57BL6/N mouse (novel mouse) and the familiar live mouse previously used in the dummy phase. This phase served as a measure of social recognition. The percent of time spent with the novel mouse was calculated by the formula (time spent with the novel mouse/(time spent with the novel mouse + time spent with the familiar mouse)) × 100 and used to assess relative exploration time between the familiar and unfamiliar congenic mouse.

### Amphetamine Sensitivity Test

An AMPH-induced locomotor hyperactivity test in the open field was used to assess the animals’ behavioral response to acute dopaminergic stimulation. The test apparatus and protocol are fully described in the Supplementary Materials. *D*-amphetamine sulfate (Sigma–Aldrich) was dissolved in isotonic 0.9% NaCl solution to achieve the desired concentration for injection. The AMPH concentration was administered via the i.p. route at a dose of 2.5mg/kg. The dose was selected based on our previous studies showing that poly(I:C) offspring display enhanced locomotor responses to AMPH at this dose (e.g., Meyer et al., 2005, 2008).

### SH-053-2’F-S-CH_3_ Preparation and Administration

A detailed description of SH-053-2’F-S-CH_3_ synthesis has been described previously (Cook et al., 2009). Synthesis of (*S*)-ethyl 8-ethynyl-6-(2-fluorophenyl)-4-methyl-4*H*-benzo[*f*]imidazo[1,5*-a*][1,4]diazepine-3-carboxylate was performed at the Department of Chemistry and Biochemistry, University of Wisconsin, Milwaukee, WI. SH-053-2′F-S-CH_3_ has been demonstrated to have a greater relative affinity for α5 (Ki = 19.2), α2 (Ki = 33.3), and α3 (Ki = 291.5) compared with the relative affinity for α1 (Ki = 468.2; Fischer et al., 2010; Savić et al., 2010). Moreover, SH-053-2’F-S-CH_3_ has been shown to have a greater efficacy at α5 (218/389), α2 (170/348), and α3 (138/301) compared with the relative efficacy at α1 (116/164; efficacy expressed as percentage of control current at 100nM and 1μM; Fischer et al., 2010; Savić et al., 2010). SH-053-2’F-S-CH_3_ was dissolved with the aid of sonication in a solvent containing 85% deionized water, 14% propylene glycol, and 1% Tween 80. Corresponding VEH solution consisted of the solvent only. SH-053-2’F-S-CH_3_ was administered at a dose of 15 or 30mg/kg (i.p.) according to dose ranges reported previously (e.g., Savić et al., 2010). All solutions were freshly prepared on the day of administration and were injected using an injection volume of 5ml/kg (i.p.).

### Statistical Analysis

All gene expression (real-time PCR) data were analyzed using independent student’s *t* tests (two-tailed). In the Y-maze working memory test, the relative time spent in the novel arm and distance moved during the choice phase were analyzed using a 2×3 (prenatal treatment × drug treatment) analysis of variance (ANOVA). The data obtained in the social interaction test were separately analyzed for the two successive phases. In the first phase, a 2×3 (prenatal treatment × drug treatment) ANOVA of the percent of time spent with the live mouse was used to assess relative exploration time between a congenic mouse and an inanimate dummy object. In the second phase, a 2×3 (prenatal treatment × drug treatment) ANOVA of the percent time spent with the unfamiliar mouse was used to assess the relative exploration time between a novel congenic mouse and a familiar congenic mouse. In the AMPH sensitivity test, the distance moved was expressed as a function of 5min bins and analyzed using a 2×3 × 6 (prenatal treatment × drug treatment × bins) repeated-measure ANOVA for the initial pre-AMPH phase, and using a 2×3 × 12 (prenatal treatment × drug treatment × bins) repeated-measure ANOVA for the subsequent AMPH phase. All ANOVAs were followed by Scheffe’s post hoc comparisons whenever appropriate. Statistical significance was set at *p* < 0.05. All statistical analyses were performed using the statistical software StatView (SAS Institute Inc., version 5.0) implemented on a PC running the Windows XP operating system.

## Results

### Effects of Prenatal Immune Activation on the Gene Expression Profile of the GABA_A_ Receptor α1–5 Subunits

**Figure 1.**
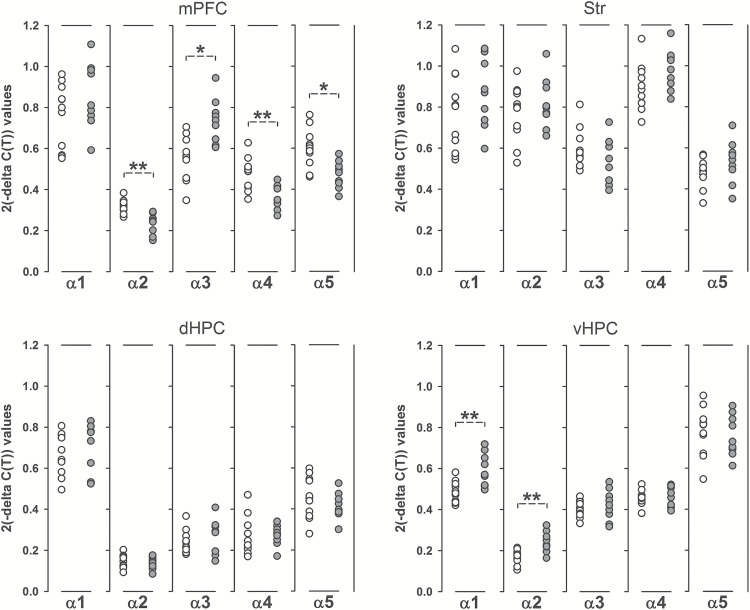
Prenatal immune activation alters the gene expression profile of the γ-aminobutyric acid (GABA_A_) receptor α1–5 subunits in the medial prefrontal cortex and ventral hippocampus. The graphs depict the individual 2(-Delta C[T]) values for the medial prefrontal cortex (mPFC), striatum (Str), dorsal hippocampus (dHPC), and ventral hippocampus (vHPC) of control (CON) and polyriboinosinic-polyribocytidilic acid–exposed (POL) offspring. CON: n = 11; POL: n = 9; for each region and subunit. *p < 0.05 and **p < 0.01. All values are means ± standard error of the mean.

We first determined the gene expression levels of the α1–5 subunits of the GABA_A_ receptor in the mPFC, Str, dHPC, and vHPC of prenatally poly(I:C)–exposed and control offspring. As summarized in Figure 1, poly(I:C) offspring displayed a significant reduction in the expression levels of the α2 (-15%; *p* < 0.01, *t*_18_ = 3.76), α4 (-20%; *p* < 0.01, *t*_18_ = 2.91), and α5 (-15%; *p* < 0.05, *t*_18_ = 2.73) subunits in the mPFC compared to controls. Moreover, prenatal poly(I:C) treatment significantly increased the expression of α3 in the mPFC by 23% (*p* < 0.05, *t*_18_ = 2.70; Figure 1). Prenatal poly(I:C) exposure did not significantly affect the expression levels of the α1–5 subunits in the Str or dHPC (Figure 1). Poly(I:C) offspring displayed, however, a significant increase in the expression levels of the α1 (+20%; *p* < 0.01, *t*_18_ = 2.93) and α2 (+25%; *p* < 0.01, *t*_18_ = 2.96) subunits in the vHPC relative to controls (Figure 1).

### Effects of SH-053-2’F-S-CH_3_ on Working Memory Deficits Induced by Prenatal Immune Activation

**Figure 2.**
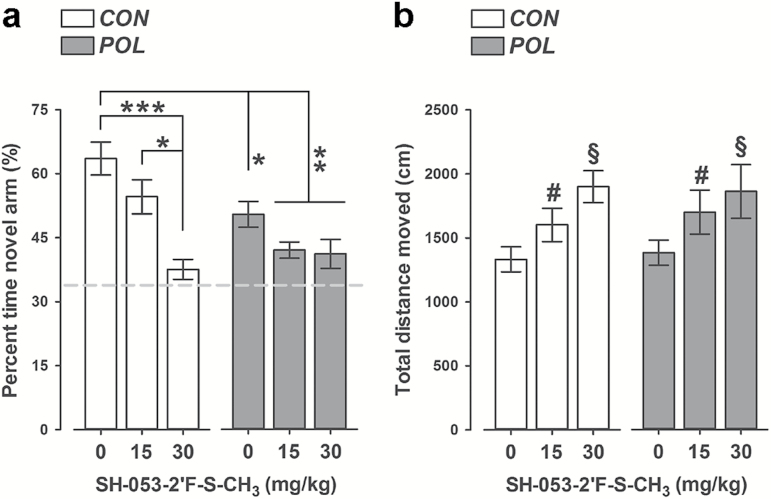
SH-053-2′F-S-CH_3_ administration produces working memory deficits in the Y-maze spatial recognition paradigm in both control and polyriboinosinic-polyribocytidilic acid [POL] offspring. (A) The bar plot depicts the percent of time spent in the novel (previously unexplored) arm during the choice phases of the test following vehicle (0mg/kg SH-053-2′F-S-CH_3_) or SH-053-2′F-S-CH_3_ (at 15 or 30mg/kg) treatment in control (CON) and POL offspring. The dashed line represents the chance level. *p < 0.05, **p < 0.01 and ***p < 0.001, based on Scheffe’s post hoc tests. (B) The graph shows the total distance moved during the choice phases of the test. ^#^ *p* < 0.01 and ^§^ *p* < 0.001, reflecting the increase in distance moved displayed by animals treated with 15mg/kg and 30mg/kg SH-053-2′F-S-CH_3_, respectively, relative to vehicle treatment; *p*-values are based on Scheffe’s post hoc tests. CON, 0mg/kg, n = 12; CON, 15mg/kg, n = 11; CON, 30mg/kg, n = 11; POL, 0mg/kg, n = 12; POL, 15mg/kg, n = 11; and POL, 30mg/kg, n = 11. All values are means ± standard error of the mean.

We evaluated possible pro-cognitive effects of SH-053-2’F-S-CH_3_ using a spatial-recognition working-memory test in the Y-maze. The critical measure of spatial-recognition memory is the relative time spent in the novel (previously unexplored) arm during the choice phase of this test. As expected, VEH-treated control offspring displayed a noticeable preference towards the novel arm, indicating intact working memory in these animals (Figure 2A). Poly(I:C) offspring exhibited a significant reduction in this measure regardless of whether they were treated with VEH or SH-053-2’F-S-CH_3_ (Figure 2A), suggesting that the PAM failed to restore working memory deficits in poly(I:C) offspring. Moreover, administration of the higher dose of SH-053-2’F-S-CH_3_ (30mg/kg) in prenatal control offspring also impaired working memory (Figure 2A), with both groups regressing to chance levels of performance after treatment with the PAM. ANOVA of relative time spent in the novel arm revealed a significant main effect of prenatal treatment [*F*(1,62) = 7.51, *p* < 0.01], drug treatment [*F*(2,62) = 14.45, *p* < 0.001], and their interaction [*F*(2,62) = 4.16, *p* < 0.05]. Subsequent post hoc analysis confirmed the significant difference between VEH-treated control and poly(I:C) offspring (*p* < 0.01); between VEH-treated control offspring and poly(I:C) offspring treated with SH-053-2’F-S-CH_3_ (*p*-values < 0.001); and between VEH-treated control offspring and control offspring treated with SH-053-2’F-S-CH_3_ at 30mg/kg (*p* < 0.001; see Figure 2A).

Prenatal poly(I:C) exposure did not significantly affect the distance moved during the Y-maze test (Figure 2B). Administration of SH-053-2’F-S-CH_3_, however, led to an increase in the total distance moved independent of the prenatal treatment histories (Figure 2B), as supported by the significant main effect of drug treatment in the ANOVA of total distance moved [*F*(2,62) = 5.67, *p* < 0.01]. Subsequent post hoc tests verified the significant difference between animals treated with VEH and SH-053-2’F-S-CH_3_ at 15mg/kg (*p* < 0.05), as well as between animals treated with VEH and SH-053-2’F-S-CH_3_ at 30mg/kg (*p* < 0.01; see Figure 2B).

### Effects of SH-053-2’F-S-CH_3_ on Social Interaction and Recognition Deficits Induced by Prenatal Immune Activation

**Figure 3.**
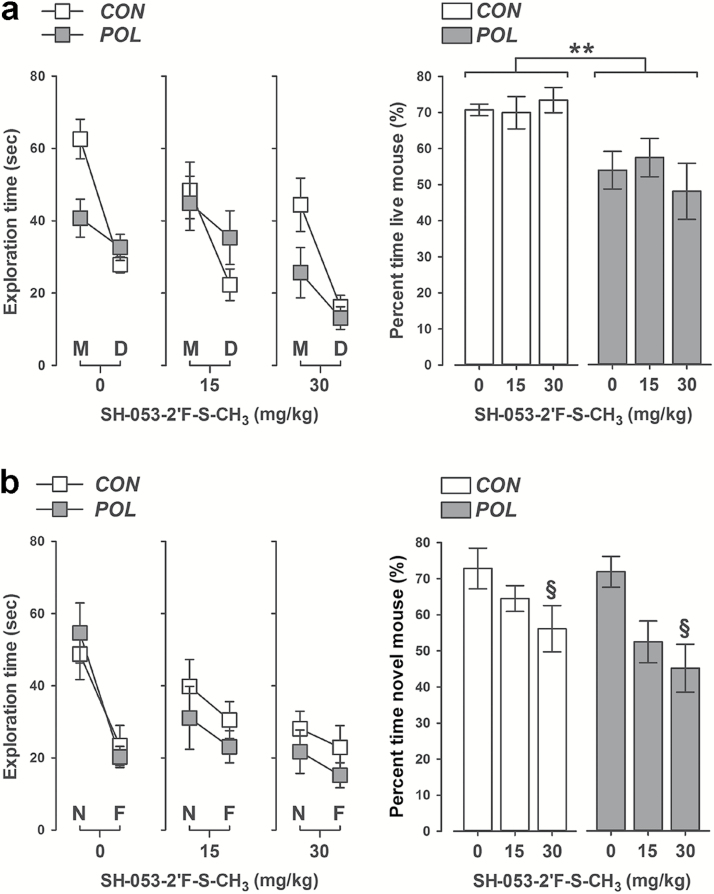
SH-053-2′F-S-CH_3_ does not impair social approach behavior but interferes with social recognition performance in both control and polyriboinosinic-polyribocytidilic acid [poly(I:C); POL] offspring. (A) Behavioral outcomes in the social approach test (dummy phase). The line plot shows absolute exploration time of an unfamiliar live mouse (M) and inanimate dummy object (D) separately for control (CON) and (POL) offspring treated with vehicle (0mg/kg SH-053-2′F-S-CH_3_) or SH-053-2′F-S-CH_3_ (at 15 or 30mg/kg). The bar plot depicts the percent time spent with the live mouse in the social approach test. *p < 0.01, reflecting the significant main effect of prenatal poly(I:C) exposure. (B) Behavioral outcomes in the social recognition test (novelty phase). The line plot shows absolute exploration time of a novel live mouse (N) and the familiar live mouse (F) separately for CON and POL offspring treated with vehicle or SH-053-2′F-S-CH_3_ (at 15 or 30mg/kg). The bar plot depicts the percent time spent with the novel mouse in the social recognition test. ^§^ *p* < 0.01, reflecting the significant decrease displayed by animals treated with 30mg/kg SH-053-2′F-S-CH_3_ relative to vehicle-treated animals. CON, 0mg/kg, n = 11; CON, 15mg/kg, n = 12; CON, 30mg/kg, n = 11; POL, 0mg/kg, n = 11; POL, 15mg/kg, n = 11; and POL, 30mg/kg, n = 12. All values are means ± standard error of the mean, and all *p*-values are based on Scheffe’s post hoc tests.

In a next step, we investigated whether SH-053-2’F-S-CH_3_ would be effective in normalizing social interaction deficits that are typically seen following a prenatal immune challenge. The relative exploration time between an unfamiliar congenic mouse and an inanimate dummy object was used to assess social approach behavior in the first phase of the social interaction test. As shown in Figure 3A, prenatal control offspring displayed a clear preference towards the unfamiliar live mouse regardless of whether they were treated with VEH or SH-053-2’F-S-CH_3_. Such social approach behavior was significantly disrupted in poly(I:C) offspring, independent of whether they received VEH or SH-053-2’F-S-CH_3_ treatment. Indeed, poly(I:C) offspring did not display a clear preference towards the unfamiliar live mouse, such that the percent of time spent with the live mouse was approximately at the 50% (chance) level in these animals (see Figure 3A). ANOVA of the percent time spent with the live mouse revealed only a significant main effect of prenatal treatment [*F*(1,62) = 7.05, *p* < 0.01].

In the second phase of the test, the relative exploration time between the previously explored live mouse and a novel unfamiliar mouse was then used to assess social recognition memory. Both control and poly(I:C) offspring treated with VEH displayed a clear preference towards the novel unfamiliar mouse relative to the previously explored mouse, suggesting that prenatal poly(I:C) exposure did not affect social recognition memory (see Figure 3B). Administration of SH-053-2’F-S-CH_3_, however, significantly impaired social recognition memory regardless of the prenatal treatment histories (Figure 3B). Indeed, the percent of time spent with the novel unfamiliar mouse decreased with increasing doses of SH-053-2’F-S-CH_3,_ leading to a significant main effect of drug treatment in the ANOVA of this measure [*F*(2,62) = 5.42, *p* < 0.01]. Subsequent post hoc analyses confirmed the significant difference between animals treated with VEH and SH-053-2’F-S-CH_3_ at 15mg/kg (*p* < 0.05), as well as between animals treated with VEH and SH-053-2’F-S-CH_3_ at 30mg/kg (*p* < 0.01; Figure 3B).

### Effects of SH-053-2’F-S-CH_3_ on AMPH Hypersensitivity Induced by Prenatal Immune Activation

Finally, we also explored whether SH-053-2’F-S-CH_3_ may be effective in normalizing potentiated AMPH sensitivity typically emerging following prenatal immune activation. For this purpose, we assessed the effects of the PAM on AMPH-induced hyperactivity in the open field test.

**Figure 4.**
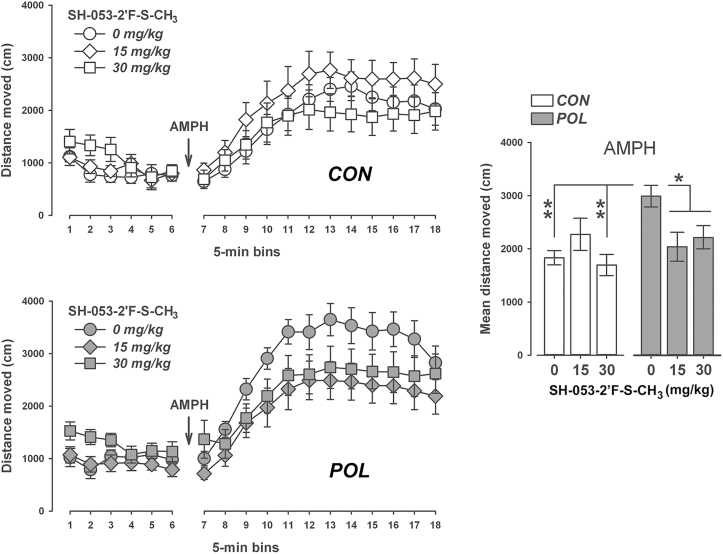
SH-053-2′F-S-CH_3_ selectively reverses the potentiated amphetamine (AMPH) locomotor response in polyriboinosinic-polyribocytidilic acid (POL) offspring. The line plots show the distance moved in the open field as a function of 5min bins during the initial habituation phase and the subsequent AMPH (2.5mg/kg, i.p.) exposure phase separately for control (CON) and (POL) offspring treated with vehicle (0mg/kg SH-053-2′F-S-CH_3_) or SH-053-2′F-S-CH_3_ (at 15 or 30mg/kg). The bar plot depicts the mean distance moved during the AMPH exposure phase for all groups. *p < 0.05 and **p < 0.01, based on Scheffe’s post hoc analyses. CON, 0mg/kg, n = 11; CON, 15mg/kg, n = 11; CON, 30mg/kg, n = 12; POL, 0mg/kg, n = 11; POL, 15mg/kg, n = 12; and POL, 30mg/kg, n = 11. All values are means ± standard error of the mean.

Neither prenatal poly(I:C) exposure nor SH-053-2’F-S-CH_3_ pretreatment significantly affected the total distance moved during the initial 30min period of the open field test (Figure 4). Poly(I:C) and control offspring treated with the higher dose of SH-053-2’F-S-CH_3_ (30mg/kg) tended to show higher locomotor activity scores during this initial testing period, but this effect did not attain statistical significance (Figure 4). ANOVA of the total distance moved only revealed a significant main effect of bins [*F*(5,310) = 9.04, *p* < 0.001], reflecting the overall changes in locomotor activity resulting from habituation to the open field.

Subsequent administration of AMPH led to a general increase in the total distance moved, as supported by the main effect of bins [*F*(11,682) = 77.78, *p* < 0.001], in the AMPH phase of the test. VEH-treated poly(I:C) offspring showed a marked potentiation of the AMPH-induced hyperactivity response compared with the locomotor-enhancing effects of AMPH in VEH-treated control offspring (Figure 4). Most interestingly, SH-053-2’F-S-CH_3_ pretreatment fully normalized the potentiation of the AMPH-induced hyperactivity response in poly(I:C) offspring, without significantly affecting the locomotor-enhancing effects of AMPH exposure in control offspring (Figure 4). These impressions were supported by ANOVA revealing a significant interaction between prenatal treatment and drug treatment [*F*(2,62) = 3.56, *p* < 0.05], and a significant three-way interaction between prenatal treatment, drug treatment, and bins [*F*(22,682) = 1.60, *p* < 0.05]. Subsequent post hoc analyses of the mean distance moved across the 60min AMPH test period confirmed a significant difference between VEH-treated control and poly(I:C) offspring (*p* < 0.05), as well as between VEH-treated poly(I:C) offspring and poly(I:C) offspring treated with SH-053-2’F-S-CH_3_ (*p*-values < 0.05).

## Discussion

The present study confirms that prenatal immune activation by the viral mimic poly(I:C) alters GABAergic gene expression in the adult central nervous system (Richetto et al., 2013, 2014). Here, we replicated our initial findings of impaired α2, α4, and α5 gene expression in the mPFC of adult poly(I:C) offspring (Richetto et al., 2014). Similar reductions in cortical α4 and α5 gene expression have been found in schizophrenia and other neurodevelopmental disorders with prenatal infectious etiologies, including autism (Duncan et al., 2010; Beneyto et al., 2011; Blatt and Fatemi, 2011). On the other hand, our findings do not parallel the reports of decreased and increased α1 and α2 mRNA levels, respectively, in the cortical areas of schizophrenia patients (Hashimoto, Arion, et al., 2008; Beneyto et al., 2011). Adult poly(I:C) offspring, however, displayed a significant increase in α1 and α2 gene expression in the vHPC, the latter being consistent with previous immunohistochemical studies showing increased α2 protein expression in the vHPC of poly(I:C)-exposed offspring (Meyer et al., 2008). Interestingly, the dHPC was largely spared by the prenatal manipulation with respect to GABA_A_ receptor alterations, and similar region-specific effects have been reported for other GABAergic markers such as parvalbumin and reelin (Meyer et al., 2008). It thus appears that the vHPC may be more susceptible to the disrupting effects of prenatal immune challenge compared to the dHPC. This notion fits well with accumulating evidence supporting a pivotal role of ventral hippocampal GABAergic abnormalities in developmental psychiatric disorders such as schizophrenia (Steullet et al., 2010; Grace, 2010, 2012; Lodge and Grace, 2011).

Based on the GABAergic effects reported here and previously (Meyer et al., 2008; Richetto et al., 2014), we speculated that administration of SH-053-2’F-S-CH_3_, a positive allosteric modulator with high affinity for the α2 and α5 (and to a lesser extent for α3) subunits of the GABA_A_ receptor, may exert beneficial effects against prenatal infection-induced behavioral abnormalities. Consistent with previous studies (Bitanihirwe, Peleg-Raibstein, et al., 2010; Bitanihirwe, Weber, et al., 2010; Connor et al., 2012; Richetto et al., 2013, 2014), poly(I:C) offspring were found to display impaired working memory in the Y-maze spatial recognition test and reduced social approach behavior in the social interaction test. Furthermore, they exhibited increased sensitivity to the locomotor-enhancing effects of the indirect dopamine-receptor agonist AMPH compared to control offspring, as reported before (Zuckerman et al., 2003; Meyer et al., 2005, 2008; Piontkewitz et al., 2011). In contrast to our hypothesis, the PAM SH-053-2’F-S-CH_3_ did not mitigate the poly(I:C)-induced working memory and social interaction deficits, two behavioral abnormalities commonly found in people with schizophrenia and related disorders (Lewis and Moghaddam, 2006; Foussias and Remington, 2010). In fact, SH-053-2’F-S-CH_3_ administration to prenatal control offspring impaired performance in the Y-maze working memory and social interaction tests. In the latter, SH-053-2’F-S-CH_3_ pretreatment led to a selective impairment in social recognition but not social approach behavior, suggesting that the drug negatively affected short-term retention of social cues rather than social approach behavior per se. Several previous studies have shown that prenatal poly(I:C)-induced cognitive deficits in rats and mice can be restored or even prevented by atypical antipsychotic drugs such as clozapine and risperidone (Zuckerman et al., 2003; Ozawa et al., 2006; Piontkewitz et al., 2009; Meyer et al., 2010). Therefore, one implication is that a normalization of prenatal poly(I:C)-induced spatial and social recognition deficits would require a modulation of neurotransmitter systems that go beyond those primarily mediated by GABA_A_ receptors. However, one clear limitation of our study is that the assessment of cognitive functions was performed using a test that is primarily based on spontaneously-motivated behavior. Additional investigations are thus warranted to extend our findings to other cognitive domains.

The detrimental effects of SH-053-2’F-S-CH_3_ on cognitive functions revealed here are in contrast to previous findings in rats suggesting that this PAM does not negatively affect spatial reference memory as assessed in the Morris water maze test (Savić et al., 2010). We have no parsimonious explanation for this discrepancy, but it could be related to differential cognitive processes involved (short-term working memory versus long-term reference memory) and/or potentially important species differences (mice versus rats). The amnesic effects of SH-053-2’F-S-CH_3_ on short-term spatial- and social-recognition memory may also seem surprising in view of previous findings indicating that GABAergic agonists acting at the α2/α3 subunits induce pro-cognitive effects (Lewis et al., 2008; Castner et al., 2010). It needs to be pointed out, however, that the pro-cognitive effects of α2/α3 agonists remain controversial (Buchanan et al., 2011). Furthermore, accumulating evidence suggests that reduced and increased activity of the α5 subunit of the GABA_A_ receptor facilitates and impairs cognitive functions, respectively (Collinson et al., 2002; Crestani et al., 2002; Dawson et al., 2006; Tan et al., 2011; Redrobe et al., 2012; Milić et al., 2013). Hence, the negative consequences of SH-053-2′F-S-CH_3_ on short-term memory revealed here may also be explained by positive modulation of the α5 subunit, given that the drug is characterized by strong α5 activity (Fischer et al., 2010; Savić et al., 2010). One clear possibility to further test this hypothesis would be to evaluate whether compounds with selective affinity for the α5 subunit would share such cognitive disruptive effects. Intriguingly, the R-isomer of the PAM used here (namely the SH-053-2′F-R-CH_3_ isomer) shows such selectivity for the α5 subunit (Cook et al., 2009; Fischer et al., 2010; Savić et al., 2010; Gill et al., 2011), and therefore, it would provide a reasonable pharmacological tool to address these issues. In such attempts, it would be important to compare various dose ranges because the S-isomer (SH-053-2′F-S-CH_3_) and R-isomer (SH-053-2′F-R-CH_3_) differ in their affinity for α5 (Cook et al., 2009; Fischer et al., 2010; Savić et al., 2010; Gill et al., 2011). Hence, administration of the S-isomer (SH-053-2′F-S-CH_3_) and R-isomer (SH-053-2′F-R-CH_3_) at the same dose may differentially influence cognitive functions due to distinct α5 affinities.

Despite the inability of SH-053-2′F-S-CH_3_ to correct prenatal infection-induced cognitive impairments, it was highly effective in mitigating AMPH hypersensitivity in adult poly(I:C) offspring without concomitant effects in prenatal control offspring. AMPH exposure can produce psychosis-like states in healthy human subjects and exacerbate existing psychoses in patients with schizophrenia (Laruelle, 2000). Moreover, potentiation of AMPH-induced dopamine release in schizophrenia patients tends to correlate with the severity of positive symptoms (Laruelle and Abi-Dargham, 1999). The efficacy of SH-053-2’F-S-CH_3_ to correct the poly(I:C)-induced potentiation of AMPH sensitivity may thus be especially relevant for attempts to establish and validate GABA-based treatments targeting positive symptoms.

Our data are also highly congruent with previous findings showing that acute treatment with a selective α5 GABA_A_-receptor PAM (namely the SH-053-2′F-R-CH_3_ isomer) can fully reverse AMPH hypersensitivity and hyperactivity of ventral midbrain dopamine neurons in the MAM-based neurodevelopmental model of schizophrenia (Gill et al., 2011). The fact that prenatal MAM-induced AMPH hypersensitivity can be normalized by a selective α5 GABA_A_-receptor PAM indicates that the beneficial effects of SH-053-2’F-S-CH_3_ revealed here may also be largely attributable to the drug’s activity at the α5 subunit. The congruent findings obtained in the prenatal poly(I:C) and MAM administration models emphasize the possibility that alterations in the adult central GABA system may represent a critical pathological convergence point for various prenatal adversities implicated in the etiology of neurodevelopmental brain abnormalities (Volk and Lewis, 2013). These models also support the hypothesis that there is a causal link between altered signaling at α subunits of the GABA_A_ receptor and the emergence of schizophrenia-relevant dysfunctions in neurodevelopmentally compromised offspring (Stan and Lewis, 2012; Volk and Lewis, 2013), at least with respect to AMPH hypersensitivity and related hyperdopaminergic functions. Consistent with this notion, reduced GABAergic signaling at the α5 subunit has previously been implicated in other dopamine-dependent behaviors: genetically-induced deficits in α5 expression impair pre-pulse inhibition of the acoustic startle reflex (Hauser et al., 2005) and selective associative learning as assessed by the latent inhibition paradigm (Gerdjikov et al., 2008).

According to a recent hypothesis (Lodge and Grace, 2011; Grace, 2012), disinhibition of the ventral hippocampus resulting from intrahippocampal impairments in GABAergic signaling could lead to a pathological hyperactivity of the (ventral) hippocampus and subsequent increase in mesoaccumbal dopamine system function. It needs to be evaluated further whether or not similar mechanisms may underlie the prenatal poly(I:C)-induced AMPH hypersensitivity. Likewise, the mechanisms by which SH-053-2′F-S-CH_3_ can ameliorate altered AMPH sensitivity in immune-exposed offspring still await exploration. Related to this, it is possible that behavioral functions critically regulated by ventral hippocampal activity could benefit from preferential α5 GABA_A_-receptor PAMs, whereas such pharmacological compounds may lack therapeutic efficacy for cognitive functions that are more directly linked to neuronal activity in the prefrontal cortex, a site with lower α5 GABA_A_ receptor expression. It is also conceivable that region-specific increases in the expression of distinct GABA_A_ receptor α subunits (e.g., increased prefrontal α3 or ventral hippocampal α2 expression) may occlude potential effects of SH-053-2’F-S-CH3, at least in offspring with immune-mediated neurodevelopmental abnormalities.

In conclusion, our study provides preclinical support for the use of benzodiazepine-positive allosteric modulators in the symptomatic treatment of AMPH hypersensitivity that emerges following (immune-mediated) neurodevelopmental disruption. Together with the recent findings obtained in the prenatal MAM administration model (Gill et al., 2011), our data suggest that positive allosteric modulation of the GABA_A_ receptor α5 subunit may be particularly useful in mitigating pathological overactivity of the dopaminergic system. At the same time, however, our study falls short in detecting possible pro-cognitive effects of PAM treatment with selective activity at the α2, α3, and α5 subunits of the GABA_A_ receptor. The lack of such pro-cognitive effects may raise concerns regarding the effective use of some types of GABA subunit selective compounds that target multiple pathological domains that involve the co-existence of psychotic, social, and cognitive dysfunctions.

## Supplementary Material

For supplementary material accompanying this paper, visit http://www.ijnp.oxfordjournals.org/

## Statement of Interest

All authors declare that they have no conflicts of interest to disclose. The present work is purely academic. During the past three years, Dr Grace has received support from Johnson & Johnson, Lundbeck, Pfizer, GSK, Puretech Ventures, Merck, Takeda, Dainippon Sumitomo, Otsuka, Lilly, Roche, Asubio, and Abbott; and Dr Riva has received research grants from Sunovion and has further received compensation as speaker/consultant for Bristol-Myers Squibb, Dainippon Sumitomo Pharma, Eli Lilly, Servier, and Sunovion.
